# Distribution of *Vibrio* species in Shellfish and Water Samples Collected from the Atlantic Coastline of South-East Nigeria

**DOI:** 10.3329/jhpn.v31i3.16822

**Published:** 2013-09

**Authors:** Onyedikachukwu A.L. Eyisi, Uchechukwu U. Nwodo, Christian U. Iroegbu

**Affiliations:** Epidemiological Research Unit, Department of Microbiology, University of Nigeria, Nsukka, Enugu State, Nigeria

**Keywords:** Atlantic coastline, Cholera, *Vibrio*, Hikojima, Sea-water, Shellfish, Nigeria

## Abstract

Crayfish, lobster, and sea-water samples collected from five fishing islands on the Atlantic coast–Bight of Biafra (Bonny)–belonging to Ibaka Local Government Area of Akwa-Ibom State of Nigeria were bacteriologically evaluated on thiosulphate citrate bile-salt sucrose (TCBS) agar for *Vibrio* load and pathotypes. Mean log_10_ *Vibrio* counts of 7.64±2.78 cfu/g (in crayfish), 5.07±3.21 cfu/g (in lobster), and 3.06±2.27 cfu/mL (in sea-water) were obtained in rainy season (June-July) while counts in the dry season (November-December) were 6.25±1.93 cfu/g, 5.99±1.54 cfu/g, and 3.84±1.78 cfu/mL respectively. The physicochemical measurements (temperature, pH, and total dissolved solutes) of the sea-water did not vary significantly in the two seasons across all five islands. *Vibrio* species isolated were *Vibrio cholerae* (both O1 and non-O1 serotypes), *V. parahaemolyticus, V. vulnificus, V. mimicus*, *and V. fluvialis.* Both Ogawa and Inaba subtypes of *V. cholerae* O1 serotype were found. In addition, the Hikojima subtype, which had not been previously reported in the region, was isolated in two samples. The results show that these *Vibrio* species are endemic in the area.

## INTRODUCTION

The interest in *Vibrio* species worldwide stems from the history and epidemiology of cholera, a severe diarrhoeal disease thought to have originated from the Far East (Asia) thousands of years ago. The spread of cholera was sea-borne at first, affecting particularly coastal towns and fishing villages. As it gained foothold within the regions, the tribal behaviours of the populations, including funeral ceremonies, movement of people within and between countries, and the use of rivers for transportation, trade, and travel as well as source of domestic water became important factors in the dissemination of the disease on a scale reminiscent of the 19^th^ century pandemics (1,2).

The long-distance spread of the disease to Europe and the Americas through the Middle East region is thought to have begun in 1817. The Ogawa serotype is reported to have arrived at the Atlantic coast of West Africa in 1868 when the first cholera outbreak was reported in the region (3,4). From September 1970, the disease invaded West African countries one after another–Guinea and Sierra Leone (28 September 1970), Liberia (October 1970), Ivory Coast (20 October 1970), Mali and Togo (both on 24 November 1970), Dahomey (16 December 1970), Upper Volta (Burkina Faso) (17 December 1970), Nigeria and Niger (27 December 1970) (1,5). The first recorded outbreak of the disease in Nigeria in 1970 was in Lagos, with three bacteriologically-confirmed cases. Since then cholera cases have been reported in various parts of the country (6,7).

*Vibrio cholerae*, the causative organism of cholera, is just one of over 33 species described in the genus *Vibrio*, among which 12 species, including *V. cholerae*, have been reported to be pathogenic to humans. Nine of these have actually been isolated in clinical conditions attributed to them (8); 8 among them have been associated with food-borne infections of the gastrointestinal tract (9). Infections due to food-borne *Vibrio* are common in Asia, including Hong Kong, and in sub-Saharan Africa, including Nigeria (6,7,10). Sea-foods and water are the major sources of infection, and *Vibrio* species have largely been isolated from these sources in the south-eastern region of Nigeria (7,11,12). It is against this background that shellfish and water samples from five fishing islands in this region, which are the major sources of shellfish sold in inland markets of Nigeria, were screened for *Vibrio* species and pathotypes.

## MATERIALS AND METHODS

### Description of the project area

The study was carried out around five fishing islands—Asia-Obufa, Obio-Iwang, Ikot-Itie-Idung, Utam-Iyata, Utam-Antai located on the Bight of Biafra (Bonny) and belonging to Ibaka LGA of Akwa-Ibom State, Nigeria. Each island has 1,000-1,500 inhabitants. All inhabitants have homes in villages in the mainland and stay in make-shift shelters with their families to engage in daily out-sea fishing activities and shellfish processing for dispatch to the inland markets. The inhabitants have no toilet facilities and defaecate at the banks or inside the sea.

### Collection of shellfish and water samples

Freshly-harvested crayfish and lobsters were purchased from fishermen as they came ashore after the night-fishing activities. These were immediately put in sterile polythene bags to avoid further contamination and conveyed to the laboratory sandwiched between ice-blocks in insulated ice-boxes.

Three sea-water samples (250 mL each) were also collected from each of the five islands as follows: using a sterilized conical flask, the sea-water samples were collected at 3 points in each island–one just at the ocean bank, the second at about 100 m from the shore, and the third at about 500 m from the shore into the sea. The conical flask was held with its mouth down and, in that position, it was submerged into depth of about 100 cm before the mouth was turned upwards under water to fill. All water samples were transported to the laboratory under the prevailing atmospheric conditions.

### Estimation of ***Vibrio*** cell count of crayfish, lobsters, and water

### Crayfish and lobster

Approximately 2.0 g of crayfish or lobster sample was taken and homogenized into smooth slurry, using a sterile pestle and mortar; 1.0 g of the homogenate was suspended first in 1.0 mL of normal saline in a 25-mL Pyrex test tube and the volume made up to 10 mL with more saline to obtain a 1% (w/v) suspension. Subsequently, the suspension of homogenized tissue was serially diluted 10-fold, down to 10^6^; then, 1.0 mL of each dilution was spread over a freshly-prepared thiosulphate citrate bile-salt sucrose (TCBS) agar (Oxoid) plate, using a sterile bent glass-rod. The plate was incubated at 37 ^o^C in a humidified incubator for 24 hours. Triplicate plates were prepared in the same way from a separate 2.0 g sample of the same batch of crayfish or lobster. After 24 hours, the incubated plates were observed for characteristic *Vibrio* colonies. Each colony, distinguished mainly by colour and size, was counted separately. Then, discrete representatives of each colony type were randomly selected for purification and characterization.

### Water

Water samples collected from different islands were analyzed for temperature and pH at the point of collection and for total dissolved solids (TDS), using a TDS meter (Korea, HM Digital Inc.), and values were expressed in parts per million (ppm). Subsequently, approximately 200 mL of each sea-water sample was filtered through a sterile 0.45-μm membrane, using a vacuum pump to facilitate the process; then, the filter unit was carefully dismantled, the membrane aseptically removed with sterile forceps and placed on TCBS agar. After incubation at 37 ^o^C for 24 hours, the plate was examined for characteristic *Vibrio* colonies which were then counted and discrete colonies randomly selected for characterization.

### Characterization of isolates suspected to be ***Vibrio***

The randomly-selected colonies were first Gram-stained to ascertain the curved rod cell morphology of *Vibrio* species before purification by three successive streaking and re-isolations on TCBS agar. The purified isolates were cultured on nutrient agar slants and stored at 4 ^o^C. From the stock culture, each isolate was assayed for oxidase, urease and indole production, motility, and fermentation of various sugars, using the method described by Cheesbrough (13). All isolates preliminarily identified to be *Vibrio cholerae* from the above assay results were serologically tested for agglutination with polyvalent O1 antiserum (ANTEC, UK). Isolates which tested positive with polyvalent O1 antiserum were further tested with anti-Ogawa and or anti-Inaba sera to differentiate them into subtypes.

## RESULTS

*Vibrio* colonies on TCBS agar appeared either yellow (showing that they are sucrose fermenters) or green or bluish-green (meaning non-sucrose fermenters). Random microscopy of not less than 50 of each colony morphology type showed that they were predominantly curved or coma-shaped Gram-negative rods, thus confirming that they were *Vibrio* strains. With this, *Vibrio* counts were obtained from crayfish, lobster, and water samples collected from all five islands in both rainy season (June-July 2009) and the dry season (November-December 2009) as shown in Table 1. The log_10_ mean *Vibrio* counts (cfu/g) were 7.64±2.78 (with range 6.29±3.82-9.26±4.42) for crayfish, 5.07±3.21 (range: 3.11±1.08−7.37±1.00) for lobsters, and 3.06±2.27 cfu/mL (range: 1.12±6.12−7.00±1.20) for sea-water in the rainy season. The values obtained for similar samples in the dry season were 6.25±1.93 (range: 5.03±1.81-8.00±0.21), 5.99±1.54 (range: 4.13±1.13-9.25±1.30) and 3.84±1.78 (range: 0.15±2.71-9.11±1.60) respectively. Water samples were collected from each Island starting at the bank, 100 m, and 500 m from the beach into the sea; the mean log_10_ *Vibrio* counts obtained were 3.84±1.76 cfu/mL (range: 0.15±2.71-9.11±1.60), 4.42±1.61 (range: 1.98±3.31−7.45±1.00), and 5.66±1.12 cfu/mL (range: 4.19±1.41-8.01±1.11) respectively (Table 2).

Further characterization of the colony morphology types showed that the yellow colonies (sucrose fermenters) were predominantly either *V. cholerae* or *V. fluvialis* while the non-sucrose fermenters were either *V. parahaemolyticus*, *V. mimicus*, or *V. vulnificus*. All five *Vibrio* species were isolated from crayfish and lobster samples screened from each of the five islands with the frequencies shown in Table 3 and 4 in both rainy (June-July) and dry (November-December) seasons. The differences in frequency of isolation of species from different islands and in the two seasons were not statistically significant (p>0.05). Table 5 shows proportions of the *Vibrio cholerae* isolates obtained from the shellfish and water samples that were O1 and non-O1 serotypes from each of the five islands both in the rainy season and in the dry season. With the exception of the crayfish samples of rainy season and the water samples (obtained both in rainy and dry seasons) in which there were preponderance of *V. cholerae* O1 serotypes over the non-O1 strains, the relative occurrence of each serotype varied with locations. The Inaba and Ogawa subtypes of the *V. cholerae* O1 serotype were found in all locations in both shellfish and water samples. The Hikojima subtype was isolated only twice.

**Table 1.** Seasonal distribution of *Vibrio* strains in samples collected from the five islands on the Atlantic coast of South-Eastern Nigeria

Island/Source of the samples	Log_10_ mean *Vibrio* count±standard deviation (SD)					
	Rainy season			Dry season		
	Crayfish	Lobster	Water	Crayfish	Lobster	Water
Asia-Obufa	6.29±3.82	4.32±9.23	2.08±2.51	7.16±1.90	9.25±1.30	4.51±1.60
Obio-Iwang	7.12±2.32	5.73±3.13	7.00±1.20	5.27±1.52	4.13±1.13	9.11±1.60
Ikot-Itie-Idung	8.10±2.11	7.37±1.00	1.92±0.52	5.81±4.20	6.00±2.13	3.11±1.15
Utam-Iyata	9.26±4.42	4.80±1.63	3.17±1.00	5.03±1.81	6.41±1.12	2.31±1.82
Utam-Antai	7.41±1.22	3.11±1.08	1.12±6.12	8.00± 0.21	4.17±2.00	0.15±2.71
Total average	7.64±2.78	5.07±3.21	3.06±2.27	6.25±1.93	5.99±1.54	3.84±1.78

**Table 2.** Distribution of *Vibrio* counts in water collected at different distances (metres) from the beach

Island/Source of samples	Log_10_ *Vibrio* count in water collected at different distances (m) from the beach			Log_10_ mean *Vibrio* count±SD
	0	100	500	
Asia-Obufa	4.51±1.60	4.90±0.25	5.18±0.33	4.86±0.73
Obio-Iwang	9.11±1.60	1.98±3.31	5.77±1.22	5.62±2.04
Ikot-Itie-Idung	3.11±1.15	7.45±1.00	8.01±1.11	6.19±1.09
Utam-Iyata	2.31±1.82	3.50±0.18	5.13±1.55	3.65±1.18
Utam-Antai	0.15±2.71	4.27±3.31	4.19±1.41	2.87±2.47
Log_10_ mean count±SD	3.84±1.78	4.42±1.61	5.66±1.12	4.64±1.50

The physicochemical analyses of sea-water from locations in the five islands showed that the temperatures ranged between 27 ^o^C and 28 ^o^C in the rainy season (June-July) and was 28 ^o^C allover in the dry season (November-December). The pH ranges were 7.4−7.6 (rainy season) and 7.5−7.6 (dry season) while the total dissolved solutes (TDS) measured 155-164 ppm in rainy season and 155-165 ppm in dry season (Table 6).

**Table 3.** Frequency of isolation of *Vibrio* species from crayfish samples collected from the five islands during the rainy and dry seasons

Island/Source of samples	No. of isolates	Distribution of *Vibrio* species (%)				
		*Vibrio cholerae*	*Vibrio fluvialis*	*Vibrio mimicus*	*Vibrio parahaemolyticus*	*Vibrio vulnificus*
Rainy season						
Asia-Obufa	137	39 (28.47)	23 (16.79)	12 (8.76)	36 (26.28)	27 (19.71)
Obio-Iwang	124	26 (20.97)	31 (25.00)	24 (19.35)	28 (22.58)	15 (12.10)
Ikot-Itie-Idung	138	23 (16.67)	19 (13.77)	39 (28.26)	31 (22.46)	26 (18.84)
Utam-Iyata	128	33 (26.61)	28 (22.58)	24 (19.35)	28 (22.58)	15 (12.10)
Utam-Antai	143	21 (14.69)	28 (19.58)	31 (21.68)	34 (23.78)	29 (20.28)
Dry season						
Asia-Obufa	149	21 (14.09)	40 (26.85)	30 (20.13)	31 (20.81)	17 (18.12)
Obio-Iwang	135	23 (17.04)	19 (14.07)	37 (27.41)	35 (25.93)	21 (15.56)
Ikot-Itie-Idung	145	21 (14.48)	28 (19.31)	35 (24.14)	41 (28.28)	20 (13.79)
Utam-Iyata	159	26 (16.35)	32 (20.13)	37 (23.27)	38 (23.90)	26 (16.35)
Utam-Antai	168	37 (16.07)	35 (20.83)	38 (22.62)	39 (23.21)	29 (17.26)
Total	1,426	270	283	307	341	225

**Table 4.** Frequency of isolation of *Vibrio* species from lobster samples collected from the five islands during the rainy and dry seasons

Island/Source of samples	No. of isolates	Distribution of *Vibrio* species (%)				
		*Vibrio cholerae*	*Vibrio fluvialis*	*Vibrio mimicus*	*Vibrio parahaemolyticus*	*Vibrio vulnificus*
Rainy season						
Asia-Obufa	129	28 (21.71)	31 (24.03)	19 (14.73)	22 (17.05)	29 (22.48)
Obio-Iwang	122	30 (24.59)	17 (13.93)	26 (21.31)	27 (22.13)	22 (18.03)
Ikot-Itie-Idung	132	19 (14.39)	23 (17.42)	32 (24.24)	29 (21.97)	29 (21.97)
Utam-Iyata	122	30 (24.59)	17 (13.93)	26 (21.31)	27 (22.13)	22 (18.03)
Utam-Antai	133	29 (21.80)	30 (22.56)	26 (19.55)	31 (23.31)	17 (12.78)
Dry season						
Asia-Obufa	138	36 (26.08)	22 (15.94)	28 (20.29)	19 (13.77)	33 (23.91)
Obio-Iwang	124	26 (20.97)	23 (18.55)	28 (22.58)	28 (22.58)	19 (15.32)
Ikot-Itie-Idung	135	19 (14.07)	31 (22.96)	30 (22.22)	29 (21.48)	26 (19.26)
Utam-Iyata	146	34 (23.29)	24 (16.44)	31 (21.23)	33 (22.60)	24 (16.44)
Utam-Antai	157	31 (19.75)	27 (17.20)	41 (26.11)	37 (23.57)	21 (13.38)
Total	1,338	282	245	287	282	242

## DISCUSSION

The shellfish (crayfish and lobster) harvested from waters of the Atlantic coast were heavily contaminated with *Vibrio* species; *Vibrio* counts were estimated to be as high as 10^4^-10^9^ cfu/g, much higher than the counts recorded by Chigbu and Iroegbu (7). Samples of the water habitat from where the shellfish was harvested were equally heavily contaminated with *Vibrio* counts reaching 10^7^–10^9^ cfu/mL in some places. This could be assumed to be the natural source of shellfish contamination. This does not preclude the chances of the crayfish and lobsters concentrating the *Vibrio* cells as filter feeders (14). Human agencies, including direct daefecation into the sea and faecally-contaminated surface water running into the sea (prevalent in the area) could be the main contributors to contamination of the sea-water itself. Direct defaecation into the sea or river was less apparent in the locations researched by Chigbu and Iroegbu (7) and could account for the wide differences in *Vibrio* counts obtained in both studies.

**Table 5.** Distribution of *Vibrio cholerae* serotypes isolated from shellfish and water samples collected from the five islands in Akwa-Ibom State, Nigeria

Island/Source of samples	Frequency of isolation of *Vibrio cholerae* serotypes								
	Crayfish			Lobster			Water		
	No. isolated	O1	Non-O1	No. isolated	O1	Non-O1	No. isolated	O1	Non-O1
Rainy season									
Asia-Obufa	39	32	7	28	27	1	13	11	2
Obio-Iwang	26	23	3	30	11	19	17	8	9
Ikot-Itie-Idung	23	23	0	19	16	3	14	7	7
Utam-Iyata	33	27	6	30	13	17	15	10	5
Utam-Antai	21	16	5	29	11	18	8	7	1
Dry season									
Asia-Obufa	21	15	6	36	19	17	16	13	3
Obio-Iwang	23	6	17	26	4	22	13	8	5
Ikot-Itie-Idung	21	9	12	19	17	2	15	11	4
Utam-Iyata	26	18	8	34	19	15	16	14	2
Utam-Antai	27	7	20	31	13	18	13	12	1

**Table 6.** Physicochemical properties of sea-water from each study area/island

Island/Source of samples	Rainy season (June-July)			Dry season (November-December)		
	pH	Temp. (^o^C)	THS (ppm)	pH	Temp. (^o^C)	THS (ppm)
Asia-Obufa	7.6	27	158	7.6	28	155
Obio-Iwang	7.4	28	163	7.6	28	165
Ikot-Itie-Idung	7.3	28	158	7.5	28	160
Utam-Iyata	7.5	27	155	7.6	28	158
Utam-Antai	7.6	28	164	7.6	28	165

TDS=Total dissolved solids

All five *Vibrio* species isolated from shellfish and sea-water of the Atlantic coast of South-Eastern Nigeria are known to be pathogenic to humans. *Vibrio cholerae* and *V. parahaemolyticus* certainly, but *V. fluvialis and V. mimicus* also, have been associated with gastroenteritis or diarrhoeal diseases. *Vibrio vulnificus*, on the other hand, has been associated with wound infection and has been mentioned in connection with bathing in the sea and handling of fish and other sea-foods. Thus, isolation of all the five pathogenic *Vibrio* species from sea-water and shellfish harvested from this habitat is of great public and environmental health significance, given that members of the fishing communities have a culture of eating the shellfish raw, handling sea-food harvests, and swimming in the sea. It is interesting to note that Chigbu and Iroegbu (7), working in the same region but in different locations, encountered only *V. cholerae, V. parahahaemolyticus* (also isolated in this study), and *V. alginolyticus* (not isolated in this study). The main difference in the two studies is that the earlier work was carried out around the Cross River estuary (around Calabar city) and in Ikom, more than 200 km from the Atlantic coastline where environmental samples were drawn from freshwater sources; the study reported here was carried out in islands located far into the sea and where human activity was constantly within the water environment. It is likely that such human activity may have influenced both *Vibrio* counts and the variety of *Vibrio* species.

Among the five *Vibrio* species isolated, *Vibrio cholerae* is the recognized benchmark of *Vibrio* pathogenicity, and it is based on what was earlier known about the epidemiology of this species that dates back to 1883 through the early 1980s. It was assumed that the human intestine was the sole reservoir of *V. cholerae* and that the organism was incapable of surviving for long in water. It was, therefore, held that infection had to be due to recent faecal contamination of drinking-water and foods. In the same thinking, humans have been thought to be the source of contamination of the sea-water and sea-foods (as mentioned earlier). This is buttressed by the culture of direct defaecation into the sea-water, a culture that prevails among fishing communities in the five islands sampled (Eyisi, personal observation). It was, however, later observed that *V. cholerae* exhibited a capacity of entering a rest-period during adverse environmental conditions (15-17). This changed all the earlier hypotheses; *V. cholerae* can apparently survive in marine waters, frequently associated with phyto- or zoo-plankton, for several months. During seasons, when the temperatures and abundance of nutrients favour bloom of planktons, the bacteria multiply greatly (18,19). This does not contradict the fact that faecally-contaminated drinking-water constitutes the main source of infection with *V. cholerae*; the water then contaminates sea-foods as earlier posited. This sufficiently explains the much-reported relative abundance of the organisms with increase in temperature (20), such as during the dry season in the tropical regions of the world (7) or the warm summer months (21). In this work, *Vibrio* counts did not vary significantly between the dry and rainy seasons, and this may just be explained by the fairly uniform temperatures, pH, and dissolved solutes observed around all five islands in both the seasons. The uniform physicochemical conditions in the two seasons would be expected at about latitude 4 ^o^N (above the equator) where the investigation was carried out. Besides, the dry season (November-December) sampling included the cool harmattan month of December, very close to the time the region may experience the last rains (early October). There may not have been enough time for a significant change in the water temperatures, pH, and nutrient concentrations (for the bloom of planktons).

Both O1 and non-O1 serotypes of *V. cholerae* were isolated from water and shellfish samples with the O1 serotype predominant at times. The latter observation was unexpected, given that in environmental samples like the ones analyzed in this work, non-O1 serotype is expected to be more abundant. The possible explanation for this unexpected distribution pattern may be that much of the O1 serotype is constantly seeded into the water environment from human sources; and the shellfish pick the *Vibrio* contaminants, particularly the O1 serotype, from the water environment. This has a further public-health implication in the area—the risk of disseminating the pathogen(s) to the inland fish markets through the contaminated harvests (processed and unprocessed).

Only the *Vibrio* O1 serotypes were further tested to determine the subtypes present. The Ogawa and Inaba subtypes were abundantly isolated. These have largely been reported even in epidemic cholera along the West coast of Africa and in South-Eastern Nigeria in particular (7,11,12). It is significant that Hikojima subtype was isolated in two locations in this work. This is probably the first time the Hikojima subtype has been reported in Nigeria. It is not certain whether this subtype got to the West African coastline from Asia as well or it genetically arose in habitat as intermediate between the Ogawa and Inaba subtypes here in the West African or Nigerian coastal waters.

## ACKNOWLEDGEMENTS

We wish to thank the community heads of the five islands for their hospitality and help when Onyedikachi Eyisi and Uche Nwodo visited to collect samples, particularly Chief Akpate of Asia Obufa Island who provided accommodation for them. We also thank their guide and interpreter Odua Godwin Etim who gave them courage to travel to the islands in speedboat in the open sea.
